# Child Presence Detection Algorithm in School Buses Based on Infrared Array

**DOI:** 10.3390/s26133982

**Published:** 2026-06-23

**Authors:** Yongjun Liu, Gaosong Li, Xuepeng Yuan, Shuai Zhang

**Affiliations:** School of Intelligent Manufacturing, Huanghuai University, Zhumadian 463000, China; li_gssls@163.com (G.L.); yxp13168@126.com (X.Y.); 20232430@huanghuai.edu.cn (S.Z.)

**Keywords:** school bus safety, child presence detection, infrared array sensor, differential algorithm

## Abstract

**Highlights:**

**What are the main findings?**

- Combining the infrared array sensors with appropriate algorithms enables detection of children left unattended in school buses.
- The optimal parameters of the child presence detection algorithm based on the infrared array sensors were experimentally determined.

**What are the implications of the main findings?**

- By measuring the background temperature, presence temperature, and real-time temperature inside a school bus, and comparing the relationship between the real-time temperature difference and the presence temperature threshold, interference caused by variations in seating positions, distances, and material properties can be effectively eliminated, thereby enabling detection of children left unattended in the school bus.
- A high-success-rate detection scheme for children left behind in school buses, combined with low-cost engineering deployment, can effectively reduce the incidence of fatalities among such children due to heatstroke.

**Abstract:**

School buses serve as the primary mode of transportation for children traveling to and from school, and their safety measures represent a critical safeguard for children’s lives. Nevertheless, incidents in which children are left unattended on school buses—due to inadequate supervision or the children’s own actions—occur with notable frequency and can lead to fatal outcomes. To mitigate or prevent such tragedies, this paper proposes an in-vehicle thermal imaging solution based on infrared array sensors, integrated with a dedicated algorithm to detect whether a child has been left behind in the school bus. The system collects background temperature, presence temperature, and real-time temperature data inside the bus using infrared array sensors. By comparing the real-time temperature difference against a predefined presence temperature difference threshold, the algorithm determines whether a child is present under the current thermal conditions. It then verifies whether the number of positive detections within a specified temperature range meets a preset presence count threshold, thereby reaching a final decision regarding child presence. Experiments identified optimal parameters: a temperature range of 26–33 °C, a double-difference threshold (ε = 1), and a presence count threshold (*P* = 4). Random testing demonstrated that the proposed technical solution and algorithm achieve an overall detection success rate of 92.5%. This study develops a low-cost, easily deployable, non-contact thermal imaging method capable of identifying forgotten children on school buses with satisfactory accuracy. By detecting retention before harm occurs, the approach enhances the safety of children traveling by school bus.

## 1. Introduction

Heatstroke resulting from children being left unattended in school buses remains a critical issue in global traffic safety. Statistical data show that in the United States alone, more than 849 children have died from being forgotten in hot vehicles since 1998, with fatalities peaking at 53 and 52 in 2018 and 2019, respectively. As the primary mode of transportation for children commuting to and from school, the safety of school buses is particularly concerning. In China, since 1998, publicly documented fatalities caused by children being left unattended in school buses total approximately 50–80 cases, resulting in over 60 deaths. These tragedies are rarely the result of malicious neglect by guardians; rather, they often occur because children may fall asleep or hide inside the vehicle, rendering them difficult to detect during routine manual checks. Moreover, in hot environments, the in-vehicle temperature can rise to lethal levels within a short period.

Currently, child presence detection technology has garnered extensive attention from both the international academic community and the industry stakeholders, with relevant regulations and standards advancing rapidly. The Passive Safety Working Group of the United Nations World Forum for Harmonization of Vehicle Regulations (UN/WP.29) has included in-vehicle child presence monitoring in the agenda for developing Global Technical Regulations (GTRs), intended to apply to vehicles of categories M1, M2, and M3. Euro NCAP has incorporated a child presence detection (CPD) scoring item to its 2025 Roadmap, encouraging original equipment manufacturers (OEMs) to equip vehicles with such systems. In April 2012, the State Council of China issued and implemented the “Regulations on the Safety Administration of School Buses”, which explicitly stipulate that accompanying personnel must verify the number of children disembarking and ensure that all students have left the vehicle before departing.

Despite the implementation of stringent regulations in various countries, accidents involving children left unattended in school buses continue to occur frequently due to factors such as human negligence and technical limitations. Consequently, the development of an intelligent detection system capable of automatically identifying children left behind in school buses holds significant theoretical value and practical importance for enhancing school bus safety and protecting children’s lives.

The key to addressing the problem of children being left unattended in school buses lies in detecting the presence, that is, achieving in-vehicle human detection. Researchers have conducted extensive studies on preventing school bus safety incidents, which can be categorized into the following types. (1) Contact sensor- and infrared sensor-based detection methods [1,2,3,4]. Pressure, weight, or capacitive sensors installed on seats cannot detect children located in non-seat areas such as aisles or under seats. Moreover, deploying sensors on every seat leads to high system and maintenance costs. Passive infrared (PIR) sensors can only detect moving children and fail when children are sitting still or sleeping. Additionally, these sensors are sensitive to temperature and environmental variations, resulting in low detection success rates. (2) Wireless communication-based detection methods [5,6,7,8,9]. Technologies such as radio frequency identification (RFID) or ultra-wideband (UWB) are used to identify children inside the vehicle. These approaches require every child to wear an RFID tag or UWB terminal. Metallic materials inside the vehicle can interfere with the signal, and signal occlusion leads to identification failure. Deployment and application costs are relatively high. (3) Vision-based detection methods [10,11,12,13]. Cameras or similar devices are employed to recognize children inside the vehicle. Video-based detection is susceptible to variations in illumination and occlusions. For example, a child lying under a seat and sleeping may not be detected. Furthermore, video-based detection raises privacy security concerns. (4) Radar-based detection methods [14,15,16,17,18]. Radar is used to monitor children inside the vehicle. Radar detection is prone to interference from clutter and non-human movements. Detection blind spots exist, and a child obscured by a seat back may not be detected. Also, the detection performance of radar deteriorates considerably when children are asleep, especially when their backs and chests face away from the radar sensor. In addition, signal attenuation at long distances between the child and the radar also degrades detection reliability. (5) In-vehicle WiFi-based modeling and detection methods [19,20,21,22,23]. WiFi signals can characterize the influence of human movement on multipath propagation within the vehicle, enabling the extraction of subtle chest movement signals. Nevertheless, this approach is vulnerable to environmental interference and external signals, resulting in poor robustness. It has limited capability to detect sleeping or occluded children. Moreover, many school buses are not equipped with WiFi devices, leading to high deployment costs. (6) Neural network, deep learning, and artificial intelligence-based detection methods [24,25,26,27,28]. These emerging approaches rely on large-scale, high-quality datasets. They suffer from poor scene transferability and adaptability, involve high thresholds for training and parameter tuning, and still require continuous research before practical deployment.

Existing CPD approaches achieve high success rates for active children and those seated upright in their seats, yet their detection performance degrades considerably for stationary children or children with diverse postures at arbitrary in-cabin positions. In contrast, the proposed technical solution in this study enables robust detection not only for moving children but also for static children under various body postures.

According to the published literature, most researchers only propose their respective algorithms with limited experimental validations to verify the feasibility and effectiveness of their schemes, with few conducting extensive benchmark experiments to quantify the practical success rate of CPD. Several studies have reported specific CPD accuracy metrics: vision-based detection method attains a success rate of 90.2% [11]; radar-based detection methods achieve success rates of 99.0% [17] and 90% [18]; WiFi-based approaches yield success rates of 96.5% [19], 99.1% [20], 85.0% [21], and 95.0% [22]; and deep learning-based detection methods reach 91.4% [24], 93.4% [26], 95.0% [27], and 95.6% [28]. Importantly, CPD success rates are highly dependent on experimental test environments. Notably, all above-mentioned high-accuracy approaches were validated using passenger cars as the test platform, with subjects restricted to fixed seated postures. None of these studies performed verification experiments targeting school bus scenarios or evaluated detection performance against children adopting varied body postures. From a theoretical perspective, passenger cars feature compact cabin space with minimal occlusion and short sensing distances between sensors and target children, naturally facilitating superior detection accuracy. In comparison, school buses have larger interior volume, longer dimensions and more intricate structural layouts, which substantially increase the challenges of in-cabin child detection.

To address the limitations of the aforementioned techniques, this paper proposes the deployment of two infrared array sensors to monitor the real-time temperature distribution inside the school bus, and develops a corresponding algorithm to determine whether a child is left unattended in the vehicle. The infrared array sensors are installed on the ceiling of the bus, and a microcontroller unit (MCU) reads the temperature data, which are then classified and stored. Algorithmic studies are conducted to detect children under different postures inside the bus, and the corresponding temperature thresholds are obtained. The proposed algorithm was validated through experiments simulating various positions and postures (sitting or lying) of a left-behind child. Experimental results demonstrate that the algorithm achieves satisfactory accuracy, enabling effective detection of children left unattended in school buses, which is of great significance for ensuring the safety of child passengers.

## 2. Materials and Methods

**Figure 1:**
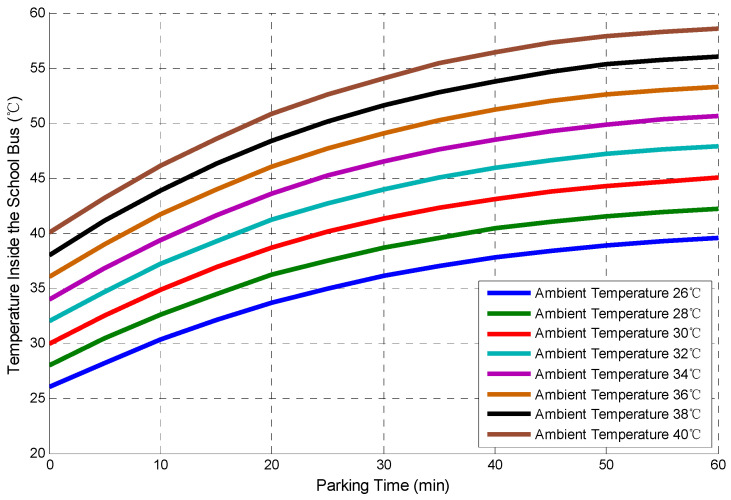
In-cabin temperature rise curves of the school bus under different ambient temperatures.

In summer, if a young child is accidentally left in a closed school bus, the in-cabin temperature rises rapidly, potentially leading to heatstroke, suffocation, organ failure, and death. Figure 1 shows the temperature rise curve inside a closed 19-seat school bus. When the ambient temperature is 30 °C, the interior temperature can approach 40 °C within 20 min. A child’s body temperature exceeding 40 °C can cause severe heatstroke, and a temperature above 42 °C poses an extremely high risk of death.

Once a child is left behind, an alarm and intervention measures must be triggered as quickly as possible. Accurate detection of the unattended child is the prerequisite for such actions, requiring high detection success rates and low false-positive and false-negative rates. Children are often left behind because they fall asleep or hide in the vehicle and thus remain motionless. In such cases, radar- and vision-based methods frequently fail to detect them. By contrast, infrared thermography, which analyzes temperature values and differences across locations and incorporates appropriate algorithms, can more effectively detect motionless children who have been left behind. Accordingly, this paper employs two infrared array sensors to monitor the real-time temperature inside the school bus and implements a detection algorithm for unattended children.

### 2.1. Sensors

#### 2.1.1. Infrared Array Sensor

Any object with a temperature above absolute zero radiates energy at specific wavelengths. Under normal temperature conditions, all objects in nature can be regarded as sources of infrared radiation, differing only in their radiation wavelengths. For example, when the human body temperature is approximately 37 °C, its infrared radiation wavelength ranges from 9 to 10 μm.

An infrared thermopile sensor converts absorbed infrared radiation into thermal energy, transforms the resulting temperature change into an electronic signal, and outputs it after amplification. As a non-contact infrared temperature sensor, the thermopile enables rapid measurement of an object’s surface temperature without direct contact. It is suitable for measuring high-temperature, hazardous, or moving objects and does not contaminate or damage the target.

By installing two infrared array sensors inside a school bus and orienting them at appropriate angles, the temperature of each seating area can be measured in real time, providing essential data for detecting children left unattended. In this study, two MLX90621 infrared thermopile array sensors (Melexis Technologies NV, Tessenderlo, Belgium) are employed to measure the in-cabin temperature.

#### 2.1.2. Sensor Layout

**Figure 2:**
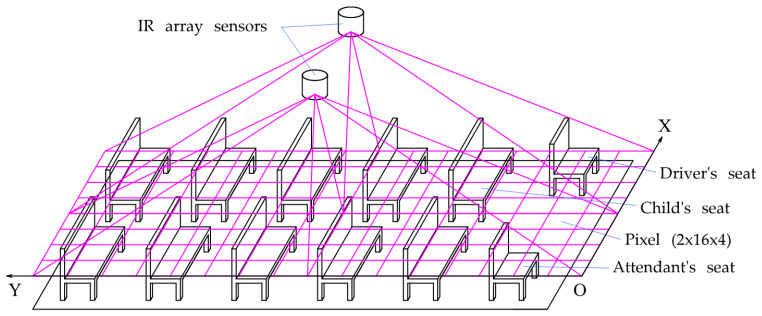
Layout of the infrared array sensors.

Most small- and medium-sized kindergarten school buses have fewer than 20 seats, with an internal length typically not exceeding 6 m and a width not exceeding 2 m. Considering wiring and cost, two infrared thermal array sensors with a resolution of 16 × 4 pixels and a field of view of 120° × 25° are selected, which can cover all seating areas, as shown in Figure 2. The two MLX90621 infrared thermopile array sensors are installed side by side on the interior ceiling of the bus. Each sensor acquires temperature values from 16 × 4 pixels, and the two sensors together collect data from 128 pixel points. Based on the acquired temperature data, a dedicated algorithm determines whether a child has been left unattended in the school bus.

### 2.2. Data Acquisition

#### 2.2.1. Circuit

**Figure 3:**
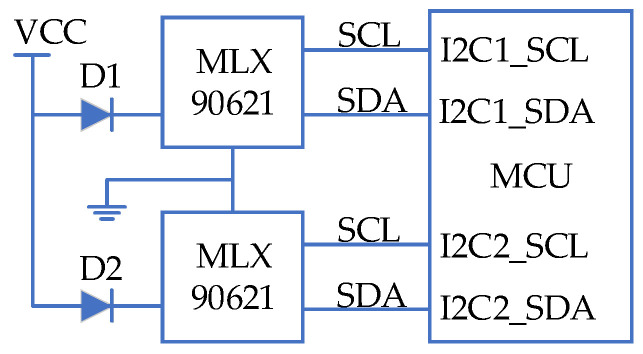
Schematic diagram of the connection between the sensors and the MCU.

The optimal supply voltage for the MLX90621 sensor is 2.6 V. In this design, a 3.3 V power supply is connected in series with a diode (voltage drop of approximately 0.7 V) to power the MLX90621 sensors. The sensors communicate with the controller via the I^2^C bus. The wiring diagram between the MLX90621 and the MCU is shown in Figure 3.

#### 2.2.2. Pixel Temperature Calculation

**Figure 4:**
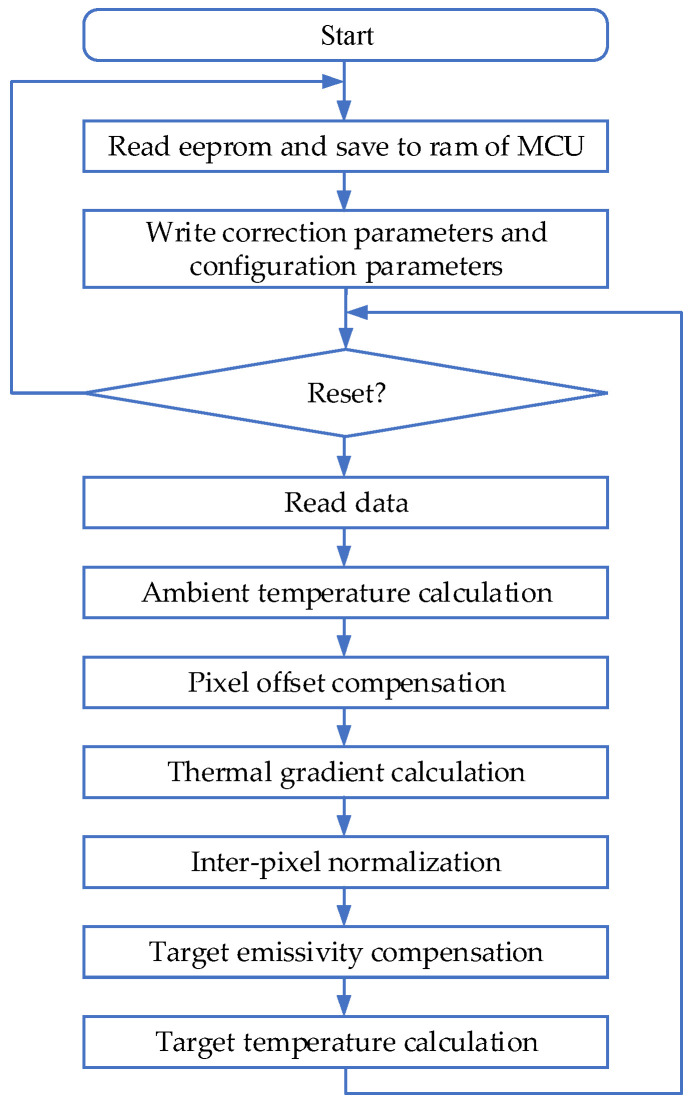
Flow chart of initialization, measurement and calculation of MLX90621.

The MLX90621 integrates an EEPROM and a RAM. The EEPROM stores chip manufacturing parameters and the parameters required for calculating pixel temperatures, while the RAM stores the measured temperature values. The initialization, measurement, and calculation processes of the MLX90621 are illustrated in Figure 4.

When using the MLX90621, it is first initialized, and then the measured values are read from the RAM via I^2^C communication. According to the device datasheet, the chip temperature *T*_a_ is calculated using Equation (1). Combined with the parameters stored in the EEPROM, the temperature of each pixel is then calculated using Equation (2).

(1) $$T_{a}=\frac{-K_{T1}+\sqrt{K_{T1}^{2}-\lambda_{1}K_{T2}(V_{TH}-PTAT)}}{\lambda_{2}K_{T2}}+T_{a0},$$

where *K*_T1_, *K*_T2_ and *V*_TH_ are parameters stored in the EEPROM, *λ*_1_ and *λ*_2_ are constant coefficients, with values of 4 and 2, respectively, and *PTAT* is a parameter stored in the RAM; all of them can be read directly from the chip. *T*_a0_ is a constant and its value is 25.

(2) $$T_{\mathrm{ij}}=\sqrt[{4}]{\frac{V_{C(i,j)}}{\alpha_{C(i,j)}*(1-K_{S4}*T_{k0})+S_{\left(i,j\right)}}+T_{\alpha K4}}-T_{k0},$$

where *T*_ij_ is the temperature of the target pixel, *V*_C(i,j)_ is the parasitic compensation parameter, *α*_C(i,j)_ is the compensation sensitivity coefficient, *K*_S4_ is the compensation factor, and *T*_k0_ is the conversion constant between thermodynamic temperature (kelvin, K) and Celsius temperature (°C), and its value is 273.15. *V*_C(i,j)_, *α*_C(i,j)_ and *K*_S4_ can be calculated from parameters readable from the EEPROM. *T*_αK4_ and *S*_(i,j)_ are intermediate conversion results, which are calculated by Equation (3) and Equation (4), respectively.

(3) $$T_{\alpha K4}={\left(T_{a}+T_{k0}\right)}^{4},$$

(4) $$S_{\left(i,j\right)}=K_{S4}*\sqrt[{4}]{{\alpha_{C(i,j)}}^{3}*V_{C(i,j)}+{\alpha_{C(i,j)}}^{4}*T_{\alpha K4}}$$

After reading the temperature of each pixel from the chip, the data can be used in the subsequent algorithm.

## 3. Algorithm

Although infrared array sensors are feasible for human detection and have been applied in practice, their use for detecting children left unattended in vehicles still faces several challenges. First, interference from complex heat sources degrades the signal-to-noise ratio. Second, the rapid change in the in-vehicle temperature field in summer may cause the human thermal radiation signal to be overwhelmed by the ambient temperature within a short period. Therefore, it is essential to develop a dedicated algorithm capable of separating the human thermal radiation signal from noise and ambient temperature fluctuations, thereby improving detection accuracy.

### 3.1. Temperature Acquisition and Storage

Factors affecting the determination of a child left unattended include the cabin ambient temperature, the cabin background temperature, and the cabin real-time temperature. These parameters must be acquired and stored.

#### 3.1.1. Cabin Ambient Temperature Acquisition

The ambient temperature refers to the air temperature inside the vehicle. It determines the temperature rise and its rate, and is a key factor causing harm to a left-behind child. The ambient temperature is acquired by a temperature sensor and denoted as *T*^(k)^ (k ∈ Z, 26 ≤ k ≤ 37). Since the normal human body temperature is approximately 37 °C, when the cabin ambient temperature exceeds 37 °C, it is no longer possible to determine whether a child is left unattended based on temperature and its differences.

#### 3.1.2. Cabin Background Temperature Acquisition

The internal structure of a school bus is complex, and temperatures at different locations (floor, seats, handrails, etc.) are not identical. A unified temperature analysis would introduce errors; therefore, the background temperature at each point must be acquired. The background temperature inside the school bus is defined as the temperature at each pixel when the vehicle is empty and the cabin ambient temperature is *T*^(k)^ (k ∈ Z, 26 ≤ k ≤ 37). The background temperature at each pixel depends on factors such as the cabin ambient temperature, the distance between the pixel and the sensor, and the material of the background. The background temperature is measured using two infrared array sensors and denoted as *T*^B(k)^_(i,j)_ (k ∈ Z, 26 ≤ k ≤ 37; i ∈ Z, 1 ≤ i ≤ 8; j ∈ Z, 1 ≤ j ≤ 16). The background temperature can be obtained experimentally and needs to be measured only once under each distinct cabin ambient temperature. If the internal structure of the school bus changes, the background temperature must be re-measured.

#### 3.1.3. Cabin Presence Temperature Acquisition

When a child is left unattended, the temperature of the pixel where the child resides is influenced by multiple factors and can be calibrated experimentally. The presence temperature is acquired using two infrared array sensors under different cabin ambient temperatures by deliberately placing a child in the vehicle and recording the temperature of the corresponding pixel at each ambient temperature. The presence temperature is denoted as *T*^P(k)^_(i,j)_ (k ∈ Z, 26 ≤ k ≤ 37; i ∈ Z, 1 ≤ i ≤ 8; j ∈ Z, 1 ≤ j ≤ 16).

Measuring the presence temperature at every pixel for each ambient temperature would be time-consuming. Taking an ambient temperature interval of 1 °C as an example, there are 12 temperature steps from 26 °C to 37 °C, and a total of 128 pixels. If a child is placed on every pixel simultaneously, the experimental difficulty is reduced, but the pixel temperatures would interfere with each other, which does not reflect actual scenarios (typically only one or two children are left behind). Conversely, if only one child is placed at a time, 1536 experiments would be required, resulting in excessive workload and cost.

Therefore, this study adopts a method that acquires the presence temperature only at several characteristic pixels and then calculates the presence temperatures at other pixels indirectly based on the background temperature gradient. As shown in Figure 2, when acquiring the presence temperature inside the bus, only four pixel positions (1,1), (1,8), (3,1) and (3,8) need to be measured. The presence temperatures at the remaining pixels are obtained by interpolation.

#### 3.1.4. Cabin Real-Time Temperature Acquisition

The real-time temperature inside the school bus is defined as the temperature value at each pixel as a function of time. The real-time temperature at each pixel is related to the cabin ambient temperature and is a key parameter for determining whether a child is present. This temperature is typically measured after the vehicle has stopped and the students have disembarked. Since child-left-behind incidents mostly occur in summer, and when the cabin ambient temperature falls below 26 °C, there is no harm to children. Therefore, real-time temperature acquisition can be initiated when the cabin temperature reaches or exceeds 26 °C. The real-time temperature at each pixel is measured and recorded by the infrared array sensors, denoted as *T*^R(k)^_(i,j)_ (k ∈ Z, 26 ≤ k ≤ 37; i ∈ Z, 1 ≤ i ≤ 8; j ∈ Z, 1 ≤ j ≤ 16).

### 3.2. Calculation of Temperature Difference

After measuring and recording the cabin ambient temperature, background temperature, and real-time temperature, the presence temperature difference threshold and the real-time temperature difference can be calculated for subsequent determination of child presence.

#### 3.2.1. Presence Temperature Difference Threshold

The presence temperature difference threshold is defined as, under a given cabin ambient temperature, the difference between the temperature of a pixel where a child is present and the temperature of the same pixel when no child is present. This threshold is experimentally measured and calculated, and serves as a criterion for determining whether a child is left unattended in the vehicle. It is denoted as Δ*T*^P(k)^_(i,j)_.

1. Calculation of the presence temperature difference threshold at special points

**Figure 5:**
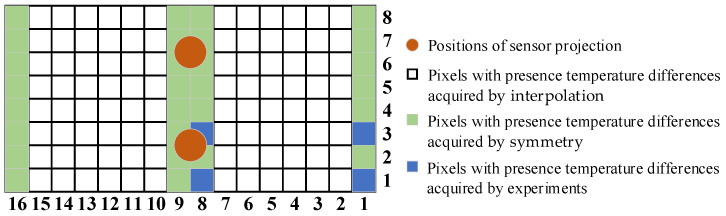
Schematic diagram for calculating the presence temperature difference threshold.

Special points refer to the pixels (1,1), (1,8), (3,1) and (3,8), as indicated by the blue pixels in Figure 5. Their presence temperatures can be obtained experimentally and the presence temperature difference thresholds can be calculated by Equation (5). The orange circular markers in Figure 5 indicate the installation positions of the infrared array sensors.

Δ*T*^P(k)^_(i,j)_ = *T*^P(k)^_(i,j)_ − *T*^B(k)^_(i,j)_, k ∈ Z, 26 ≤ k ≤ 37; (i,j) = (1,1), (1,8), (3,1), (3,8), (5)

2. Calculation of the presence temperature difference threshold at other points

Measuring the presence temperature difference threshold at each pixel under every ambient temperature would be excessively laborious. Therefore, an interpolation method is adopted. After obtaining the thresholds at the four blue pixels, and given that the infrared array sensors are installed directly above the intersection of the four pixels (2,8), (3,8), (2,9) and (3,9), the thresholds at the green pixels in Figure 5 can be directly derived from those at the blue pixels by symmetry. Specifically, for any k = 26, 27, …, 37 and for green pixel positions (i,j) with i = 1, 2, …, 8 and j = 1, 8, 9, 16, Δ*T*^P(k)^_(i,j)_ can be directly obtained from the thresholds at the corresponding blue pixel positions (i,j) = (1,1), (1,8), (3,1), (3,8). The thresholds at the remaining white pixels must be calculated by interpolation.

Since temperature and distance approximately follow an exponential relationship, the temperature measurement and its difference decrease as the distance from the sensor increases. However, the fluctuation amplitude of the temperature difference is much smaller than that of the temperature itself. To simplify the calculation, this paper adopts a linear interpolation method to compute the presence temperature difference thresholds for the following pixels:

Δ*T*^P(k)^_(i,j)_, k ∈ Z, 26 ≤ k ≤ 37; i ∈ Z, 1 ≤ i ≤ 8; j ∈ Z, 2 ≤ j ≤ 7.

Δ*T*^P(k)^_(i,j)_, k ∈ Z, 26 ≤ k ≤ 37; i ∈ Z, 1 ≤ i ≤ 8; j ∈ Z, 10 ≤ j ≤ 15.

Taking Δ*T*^P(k)^_(i,j)_ (k ∈ Z, 26 ≤ k ≤ 37; i = 1; j ∈ Z, 2 ≤ j ≤ 7) as an example, after obtaining Δ*T*^P(k)^_(1,1)_ and Δ*T*^P(k)^_(1,8)_ experimentally, the presence temperature difference thresholds for the remaining pixels in the same row can be calculated, as shown in Equation (6).

Δ*T*^P(k)^_(i,j)_ = Δ*T*^P(k)^_(i,1)_ + (j − 1) * (Δ*T*^P(k)^_(i,8)_ – Δ*T*^P(k)^_(i,1)_)/7, k ∈ Z, 26 ≤ k ≤ 37; i ∈ Z, 1 ≤ i ≤ 8; j ∈ Z, 2 ≤ j ≤ 7 (6)

Based on the symmetry principle, the values of Δ*T*^P(k)^_(i,j)_ (k ∈ Z, 26 ≤ k ≤ 37; i ∈ Z, 1 ≤ i ≤ 8; j ∈ Z, 10 ≤ j ≤ 15) can be obtained. At this point, the presence temperature difference thresholds for all pixels have been calculated.

#### 3.2.2. Real-Time Temperature Difference

Under a given cabin ambient temperature, when a child is left unattended in the school bus, the temperature of the pixel where the child resides differs from the background temperature. This difference varies with the ambient temperature and the child’s position. Generally, the difference between the real-time temperature and the background temperature at a pixel with a child present is greater than that at a pixel without a child. Therefore, the difference Δ*T*^R(k)^_(i,j)_ between the real-time temperature and the background temperature under different ambient temperatures can be recorded, as shown in Equation (7), and this real-time temperature difference can be compared with the presence temperature difference threshold as an important basis for determining whether a child is left unattended.

Δ*T*^R(k)^_(i,j)_ = *T*^R(k)^_(i,j)_ – *T*^B(k)^_(i,j)_, k ∈ Z, 26 ≤ k ≤ 37; i ∈ Z, 1 ≤ i ≤ 8; j ∈ Z, 1 ≤ j ≤ 15 (7)

### 3.3. Child Presence Determination

Whether a child is left unattended in a school bus cannot be determined simply by measuring pixel temperatures and checking whether they exceed the cabin ambient temperature. Due to the complex internal structure of the bus, pixel temperatures vary considerably. In particular, in areas made of strongly heat-absorbing materials, even without a child present, the pixel temperature may be significantly higher than the ambient temperature, leading to false positives. To address this issue, this paper adopts a method that determines child presence by checking whether the temperature rise at each pixel exceeds its presence temperature difference threshold. Specifically, under a given cabin ambient temperature, the real-time temperature difference Δ*T*^R(k)^_(i,j)_ is obtained by subtracting the background temperature (the temperature of that pixel under the same ambient temperature when no child is present) from the real-time pixel temperature. This value is then compared with the presence temperature difference threshold Δ*T*^P(k)^_(i,j)_ (i.e., the difference between the pixel temperature with a child present and the background temperature under the same ambient temperature). When Δ*T*^R(k)^_(i,j)_ is close to Δ*T*^P(k)^_(i,j)_, the presence of a child is determined, as expressed in Equation (8).

*C*^(k)^_(i,j)_ = Δ*T*^R(k)^_(i,j)_ – Δ*T*^P(k)^_(i,j)_ (8)

When the value of *C*^(k)^_(i,j)_ is significantly greater than 0, it indicates that at ambient temperature k, the temperature of pixel (i,j) is much higher than the human body temperature. This can be attributed to a strong heat source, such as a hot water bottle carried by a child, and it is determined that no child is present. When the value of *C*^(k)^_(i,j)_ is significantly less than 0, it indicates that at ambient temperature k, the temperature of pixel (i,j) is much lower than the human body temperature, which can be considered as background temperature, and it is determined that no child is present. When the value of *C*^(k)^_(i,j)_ is close to 0, it indicates that at ambient temperature k, the temperature of pixel (i,j) is close to the temperature corresponding to the presence of a child, and it is determined that a child is present. To represent how close *C*^(k)^_(i,j)_ is to 0, a small positive number *ε* is introduced, as shown in Equation (9). *ε* is defined as a double-difference threshold. If the threshold *ε* is assigned an excessively small value, the system may fail to identify children present in the cabin, raising the probability of false-negative results. Conversely, an overly large *ε* will lead to an increased false-positive rate. Hence, a properly calibrated *ε* is essential for attaining high detection accuracy.

(9) $$C^{\left(k\right)}=\left\{\begin{array}{cc} 1 & \exists {C^{\left(k\right)}}_{\left(i,j\right)}\in [-\varepsilon ,\varepsilon ]\\ 0 & \mathrm{otherwise} \end{array}\right.$$

*C*^(k)^ represents the judgment result at ambient temperature k. As long as the value of *C*^(k)^_(i,j)_ for any single pixel falls within the range [−ε, +ε], a child is considered to be present, i.e., *C*^(k)^ = 1.

When a child is left unattended, the child’s position or posture may change, causing some of the 12 *C*^(k)^ values to satisfy the child presence criterion while others do not. To address this, an auxiliary condition can be added: if more than *P* of the 12 determination outcomes are satisfied, a child is considered present, where *P* is defined as the presence count threshold. The child presence determination condition is then given by Equation (10).

(10) $$\left(\sum_{k\in Z,k=26}^{37}C^{\left(k\right)}\right)\geq P$$

### 3.4. Child Presence Detection Process

#### 3.4.1. Measurement of Background Temperature Under Different Ambient Temperatures

**Figure 6:**
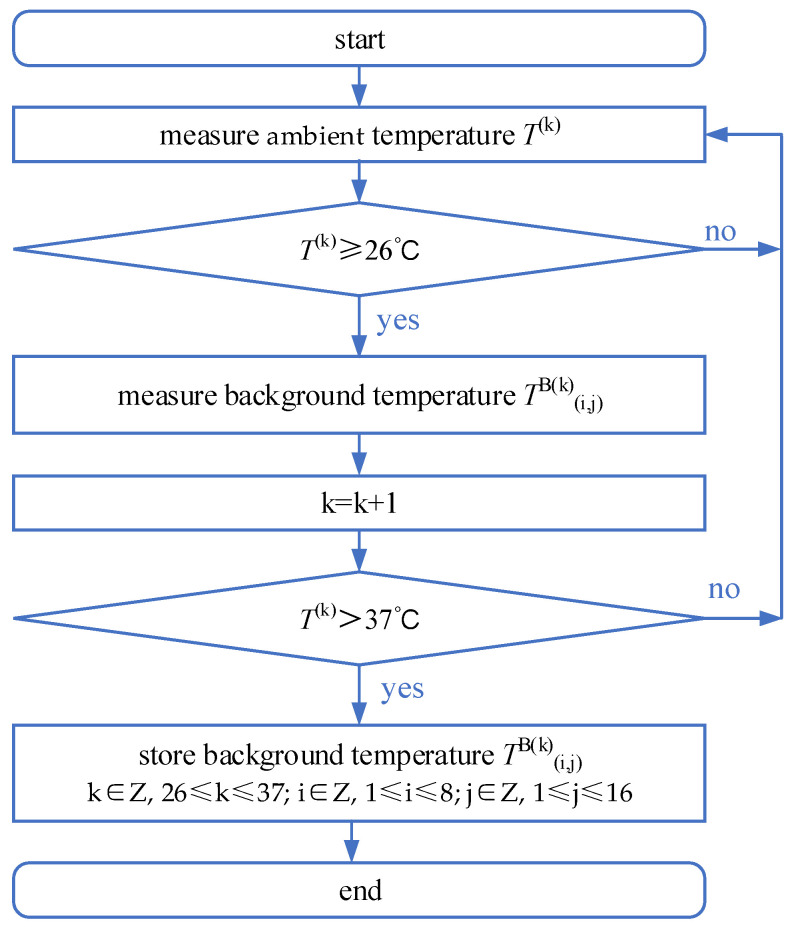
Background temperature measurement procedure.

The internal temperature field distribution varies among school buses due to differences in their internal structures and materials. Therefore, before a school bus is put into service, the background temperature under different ambient temperatures should be measured to provide fundamental data for subsequent calculation and determination. The measurement procedure for the background temperature is shown in Figure 6, and it needs to be measured only once.

I. Measure the cabin ambient temperature *T*^(k)^ using a temperature sensor installed inside the school bus.
II. If *T*^(k)^ < 26 °C, return to Step I; if *T*^(k)^ ≥ 26 °C, proceed to Step III.
III. Record the background temperature *T*^B(k)^_(i,j)_ of each pixel of the infrared array sensors under the current ambient temperature *T*^(k)^.
IV. Wait for the cabin ambient temperature to rise by 1 °C, i.e., set k = k + 1.
V. Determine whether *T*^(k)^ exceeds 37 °C: if not, return to Step I; otherwise, proceed to Step VI.
VI. Save all background temperature data *T*^B(k)^_(i,j)_, where k ∈ Z, 26 ≤ k ≤ 37; i ∈ Z, 1 ≤ i ≤ 8; j ∈ Z, 1 ≤ j ≤ 16. End of procedure.

#### 3.4.2. Calculation of Presence Temperature Difference Thresholds for Different Pixels Under Different Ambient Temperatures

**Figure 7:**
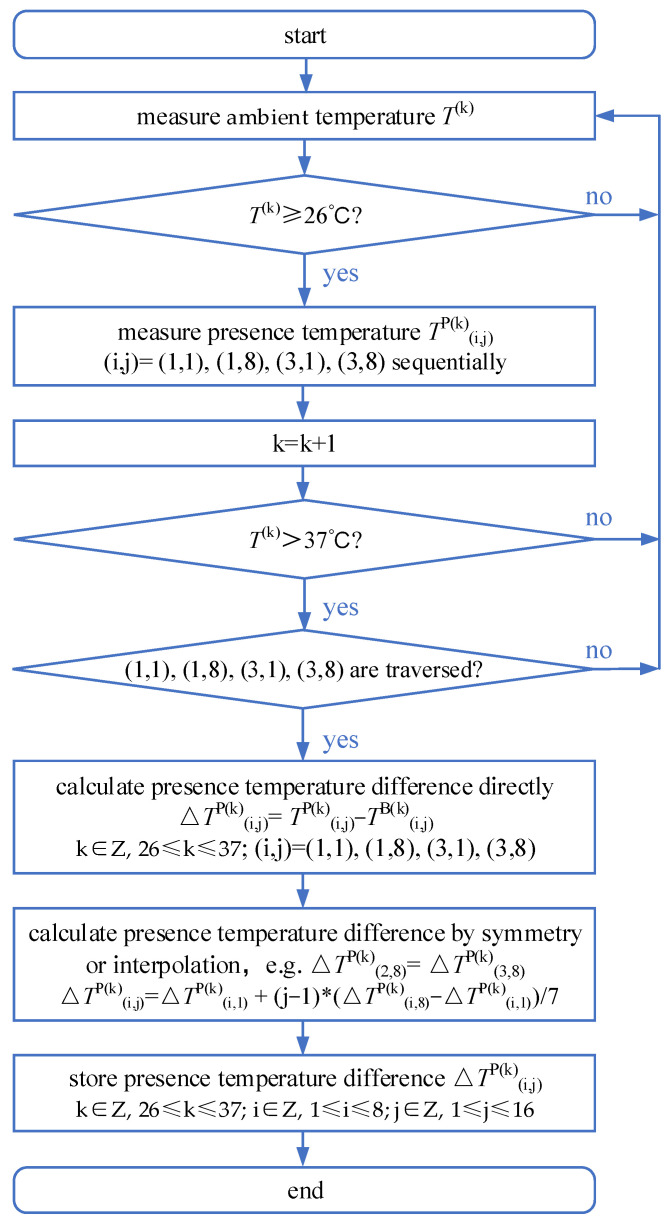
Presence temperature difference threshold calculation.

The presence temperature difference threshold is used to determine whether a child is left unattended. This threshold is obtained through a combination of limited experimental data, data symmetry, and interpolation methods, yielding the temperature difference for each pixel between the scenarios with and without a child present under different ambient temperatures. The above data need to be obtained experimentally or computationally only once and are stored in memory. The calculation procedure is shown in Figure 7.

I. Measure the cabin ambient temperature T^(k)^ using a temperature sensor installed inside the school bus.
II. If *T*^(k)^ < 26 °C, return to Step I; if *T*^(k)^ ≥ 26 °C, proceed to Step III.
III. Under the current ambient temperature *T*^(k)^, create a child presence at pixel (i,j) = (1,1) and record the presence temperature *T*^P(k)^_(i,j)_ at that pixel.
IV. Wait for the cabin ambient temperature to rise by 1 °C, i.e., set k = k + 1.
V. Determine whether *T*^(k)^ exceeds 37 °C: if not, return to Step I; and in each subsequent cycle, sequentially set the pixel to (1,8), (3,1), (3,8); if yes, proceed to Step VI.
VI. Determine whether all four pixels (1,1), (1,8), (3,1) and (3,8) have been traversed: if not, return to Step I; if yes, proceed to Step VII.
VII. Directly calculate the presence temperature difference thresholds at the four characteristic pixels.
VIII. Calculate the presence temperature difference thresholds for the following pixels by symmetry:

Δ*T*^P(k)^_(i,j)_, k ∈ Z, 26 ≤ k ≤ 37; i ∈ Z, 1 ≤ i ≤ 8; j = 1,8,9,16.

Calculate the thresholds for the following pixels by interpolation:

Δ*T*^P(k)^_(i,j)_, k ∈ Z, 26 ≤ k ≤ 37; i ∈ Z, 1 ≤ i ≤ 8; j = 2,3,…,7.

Δ*T*^P(k)^_(i,j)_, k ∈ Z, 26 ≤ k ≤ 37; i ∈ Z, 1 ≤ i ≤ 8; j = 10,11,…,15.

IX. Save all Δ*T*^P(k)^_(i,j)_ data, where k ∈ Z, 26 ≤ k ≤3 7; i ∈ Z, 1 ≤ i ≤ 8; j ∈ Z, 1 ≤ j ≤ 16. End of procedure.

#### 3.4.3. Determination of Child Presence

**Figure 8:**
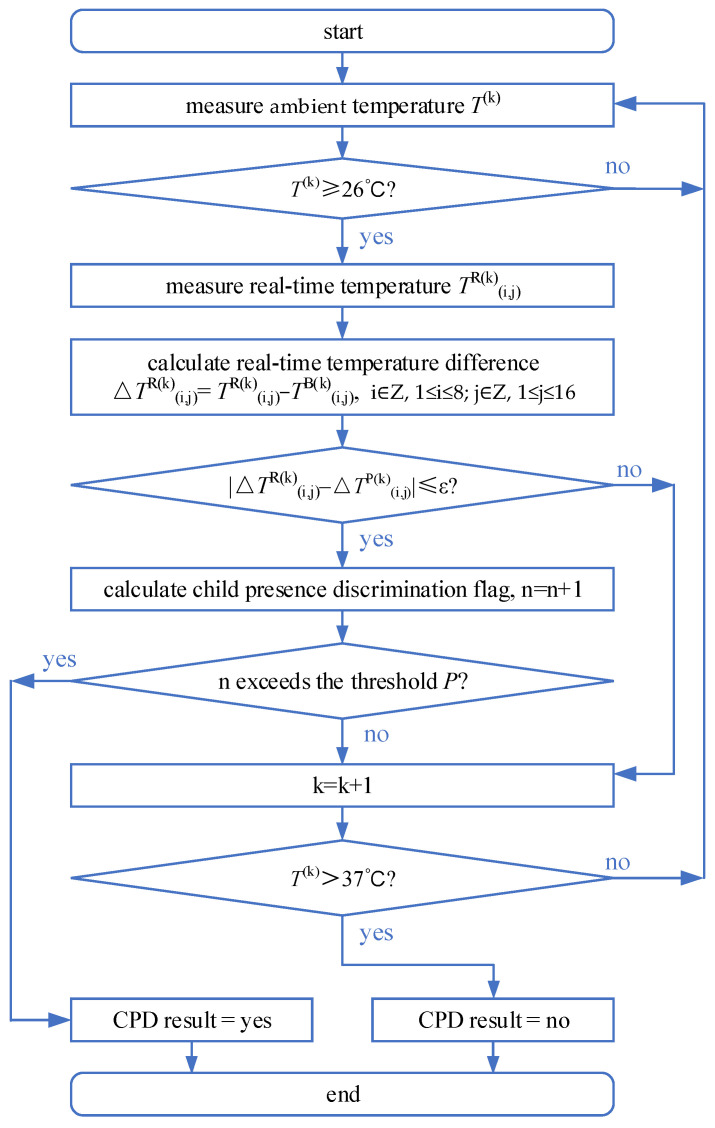
Child presence detection result determination.

Determining whether a child is left unattended in the school bus is the final output of the proposed algorithm. The core step is to compare, at each ambient temperature *T*^(k)^, the difference between the real-time temperature difference and the presence temperature difference threshold. If the two are close, it is determined that a child may be present at that ambient temperature. To improve the reliability of the determination, when the presence of a child is determined at multiple ambient temperatures *T*^(k)^, the final output is positive. The calculation procedure is shown in Figure 8.

I. Measure the cabin ambient temperature *T*^(k)^ using a temperature sensor installed inside the school bus.
II. If *T*^(k)^ < 26 °C, return to Step I; if *T*^(k)^ ≥ 26 °C, proceed to Step III.
III. Under the current ambient temperature *T*^(k)^, record the real-time temperature *T*^R(k)^_(i,j)_ of each pixel (i,j) of the infrared array sensors.
IV. Calculate the real-time temperature difference:

Δ*T*^R(k)^_(i,j)_ = *T*^R(k)^_(i,j)_ − *T*^B(k)^_(i,j)_, k ∈ Z, 26 ≤ k ≤ 37; i ∈ Z, 1 ≤ i ≤ 8; j ∈ Z, 1 ≤ j ≤ 16.

V. Check whether|Δ*T*^R(k)^_(i,j)_ – Δ*T*^P(k)^_(i,j)_| ≤ ε holds. If yes, proceed to Step VI; otherwise, go to Step VIII.
VI. Increment the detection flag (initial value 0) by 1.
VII. Check whether the detection flag exceeds the preset threshold *P*. If not, go to Step VIII; if yes, proceed to Step X.
VIII. Wait for the cabin ambient temperature to rise by 1 °C, i.e., set k = k + 1.
IX. Determine whether *T*^(k)^ exceeds 37 °C. If not, return to Step I; if yes, proceed to Step X.
X. Output the final determination (child present or no child present). End of procedure.

## 4. Case Study

To validate the effectiveness of the proposed child presence detection algorithm, a 19-seat school bus was used as the experimental platform in this study. The background temperatures and presence temperature were measured under various cabin ambient temperatures. The algorithm was then applied for child presence detection, and the detection results were compared with actual child occupancy conditions.

### 4.1. Experimental Methods and Procedures

#### 4.1.1. Experiment Conditions

The tests consist of four groups of experiments: background temperature acquisition experiments, presence temperature acquisition experiments, temperature acquisition experiments for parameter optimization, and random temperature acquisition experiments. All tests were conducted under identical experimental conditions, which are specified as follows:

(1) A 19-seat school bus for children was used as the test vehicle, with uniform seat materials and unmodified interior structures.
(2) Two infrared thermal array sensors were mounted on the vehicle ceiling, whose field of view covers all seats inside the bus.
(3) The vehicle interior was kept clean and tidy, free of extra heat sources such as pets, electronic devices and thermoses.
(4) The ambient temperature was maintained above 30 °C with relatively stable fluctuations.
(5) The air conditioning system of the school bus worked well for cooling.
(6) The school bus was parked outdoors.
(7) All children recruited for the trials were between 4 and 6 years of age, with heights ranging from 100 to 126 cm and weights from 15 to 22 kg.

#### 4.1.2. Experiment Procedure

The procedures of the four experiments are as follows.

(1) Background temperature acquisition experiment. (a) The vehicle air conditioner was activated to stabilize the cabin temperature at 26 °C for a certain duration. (b) The air conditioner was then shut down. During the in-vehicle temperature rise from 26 °C to 37 °C, temperature data output by the two infrared thermal array sensors were recorded and stored at each 1 °C temperature step. Only one round of this background temperature acquisition experiment was carried out.
(2) Presence temperature acquisition experiment. (a) The school bus air conditioner was turned on to stabilize the cabin temperature at 26 °C for a certain period. (b) One child subject was positioned at the pixel coordinate (1,1) and kept stationary at this spot throughout the test. (c) The air conditioner was switched off. While the cabin temperature increased from 26 °C to 37 °C, temperature data from the two infrared thermal array sensors were recorded and stored at every 1 °C rise. (d) Steps (a), (b) and (c) were repeated to collect and store temperature data at pixel coordinates (1,8), (3,1), and (3,8) following the identical procedure. Only one set of this presence temperature acquisition experiment was conducted.
(3) Temperature acquisition experiments for parameter optimization. (a) The air conditioning system of the school bus was activated to stabilize the cabin temperature at 26 °C for a certain period of time. (b) A single child subject was placed at a designated pixel coordinate and remained stationary at this position. (c) As the cabin temperature rose from 26 °C to 37 °C, temperature data output by the two infrared thermal array sensors were recorded and stored at each 1 °C increment. Only one trial of this temperature acquisition experiment for parameter optimization was performed.
(4) Random temperature acquisition experiment. (a) The school bus air conditioner was turned on to stabilize the cabin temperature at 26 °C for a certain duration. (b) One child subject entered the bus and freely chose the seating position and body posture. (c) As the cabin temperature increased from 26 °C to 37 °C, temperature data from the two infrared thermal array sensors were recorded and stored at every 1 °C increment. This random temperature acquisition experiment was repeated for 40 independent trials.

The background temperature data of each pixel at all ambient temperatures ranging from 26 °C to 37 °C can be directly acquired from the background temperature acquisition experiment. The presence temperature data of several specific pixels under each temperature condition (26–37 °C) can be obtained via the presence temperature acquisition experiment. After acquiring the background temperature data for all pixels and the presence temperature data for partial pixels, the presence temperature difference threshold values of all pixels can be calculated by direct subtraction, symmetry operation and interpolation methods. The differences between the temperature data collected from the temperature acquisition experiment for parameter optimization and the background temperature data are compared with the presence temperature difference thresholds to determine the optimal values of the temperature range, double-difference threshold (ε), and presence count threshold (P). With these optimal parameters obtained, the data from the random position and posture temperature acquisition experiment combined with the proposed algorithm in this paper are used to generate the final CPD results, so as to verify the feasibility and accuracy of the algorithm.

**Figure 9:**
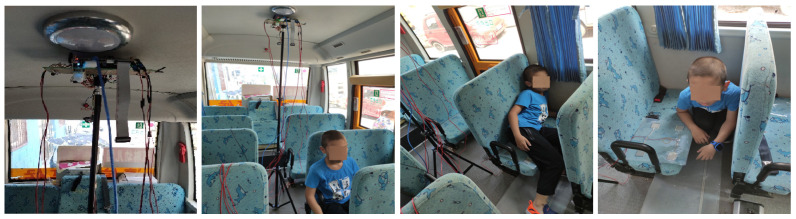
Experimental setup.

The vehicle, sensors, and child used in the experiments are shown in Figure 9.

### 4.2. Experimental Results

#### 4.2.1. Background Temperature Measurement

**Figure 10:**
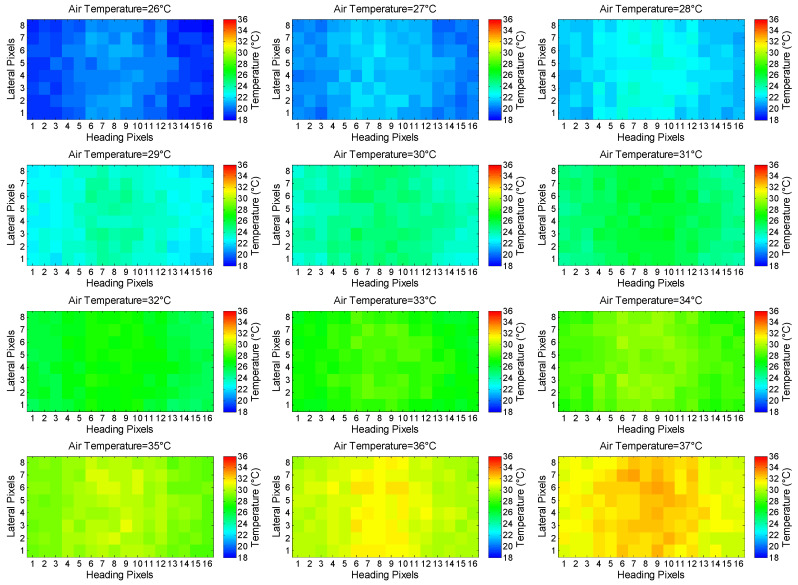
In-vehicle background temperature at different ambient temperatures.

The background temperature measurement results are shown in Figure 10. As the distance increases, the temperature gradually decreases; however, the relationship between temperature and distance is not linear, and the temperature values exhibit fluctuations. This is mainly attributed to factors such as the distance from the sensor to the measured pixel, the material of the measured pixel, and the cleanliness of the air.

#### 4.2.2. Presence Temperature Difference Threshold Calculation When a Child Is Present

**Figure 11:**
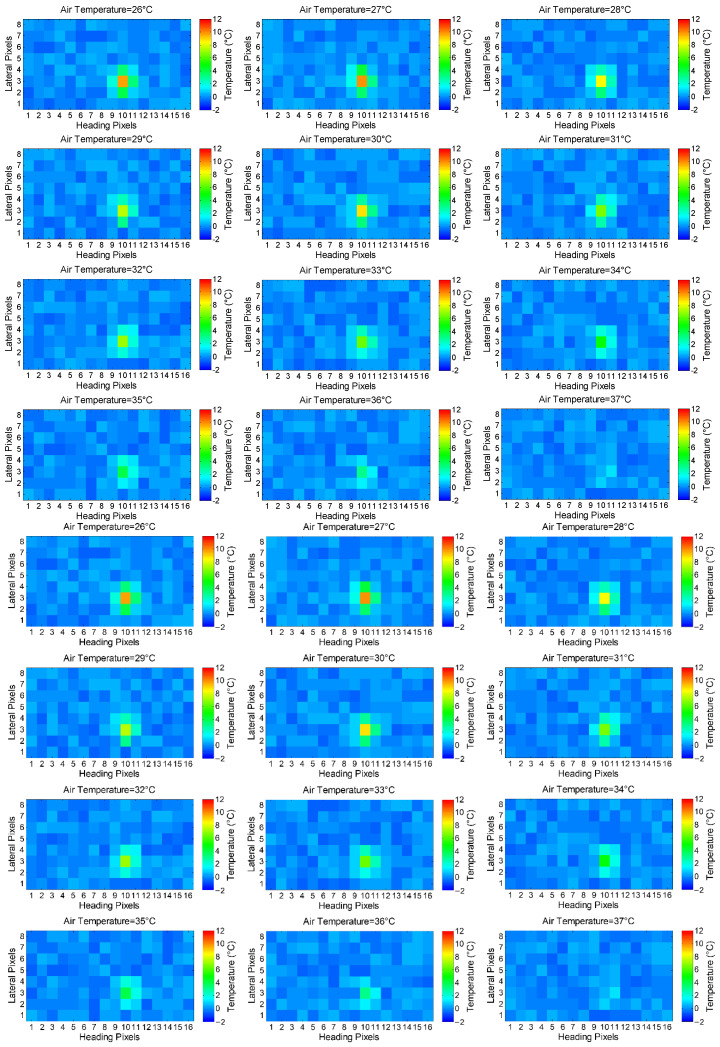
Presence temperature difference threshold under different ambient temperatures.

When a child is present inside the school bus, the presence temperature of each pixel is subtracted from the background temperature to obtain the presence temperature difference threshold at each pixel. The results are shown in Figure 11. It can be observed from the figure that (1) the presence temperature difference threshold can be used to approximately identify pixels where a child is suspected to be present; (2) when the ambient temperature is low, the presence temperature difference threshold at pixels with a child present is significantly higher than that at pixels without a child. However, as the ambient temperature increases, this difference threshold gradually diminishes and eventually disappears.

#### 4.2.3. Child Presence Detection Results

The value of *ε* plays an important role in the performance of the CPD algorithm. In order to identify the optimal *ε*, the algorithm is executed with a range of *ε* values, and the best value is determined by comparing the detection accuracy across these different settings.

**Figure 12:**
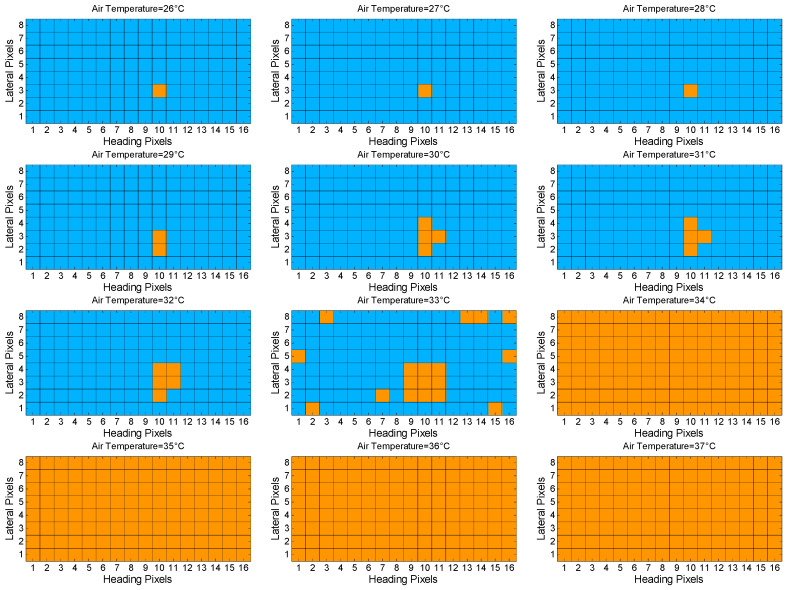
CPD results for a child left in school bus (sitting posture, ε = 5).

**Figure 13:**
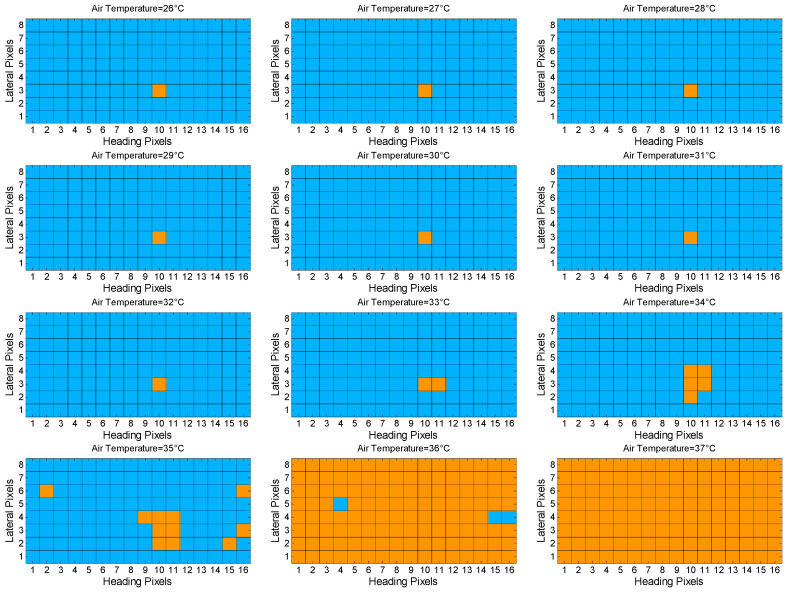
CPD results for a child left in school bus (sitting posture, ε = 3).

**Figure 14:**
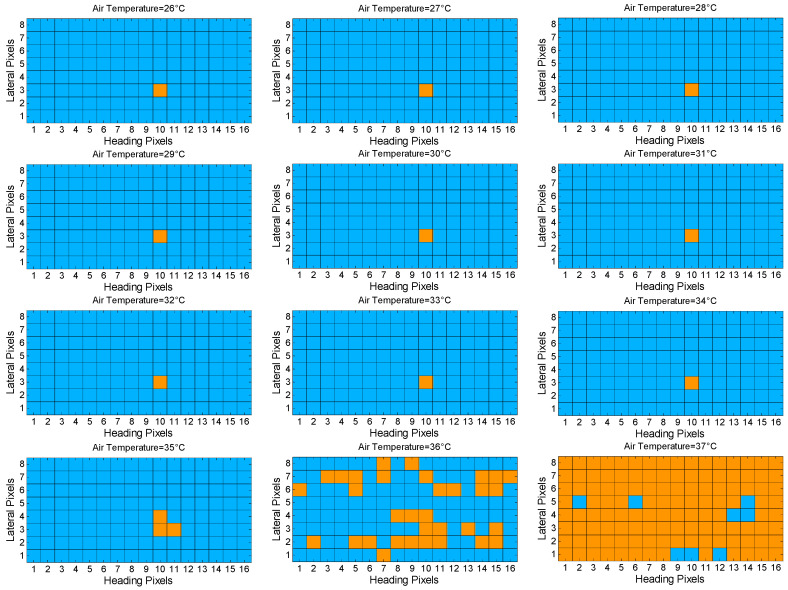
CPD results for a child left in school bus (sitting posture, ε = 2).

**Figure 15:**
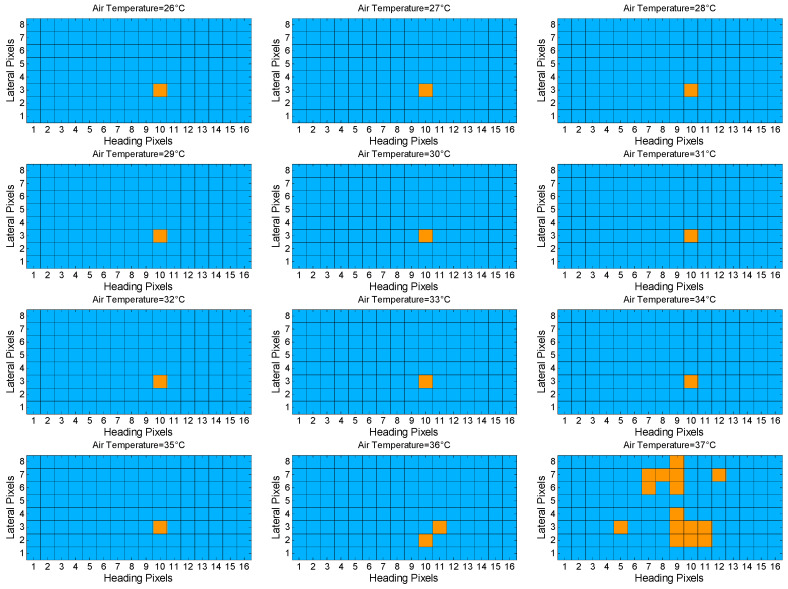
CPD results for a child left in school bus (sitting posture, ε = 1).

**Figure 16:**
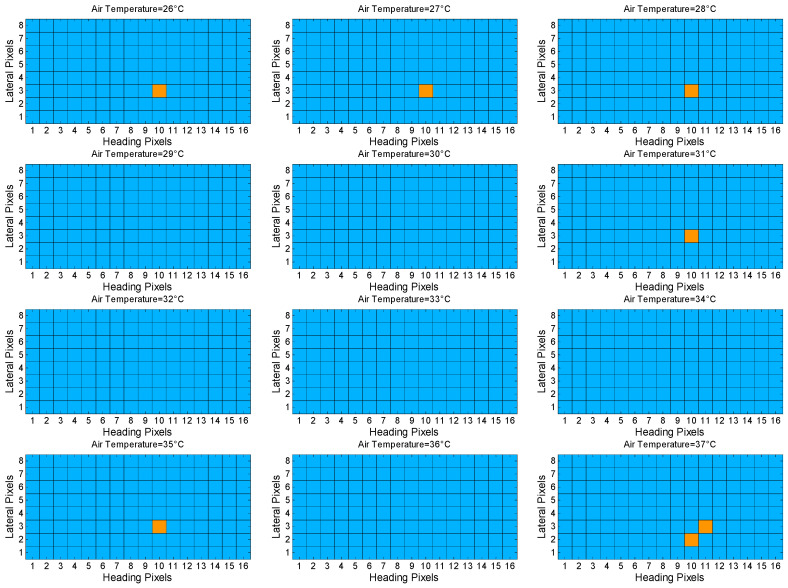
CPD results for a child left in school bus (sitting posture, ε = 0.5).

**Figure 17:**
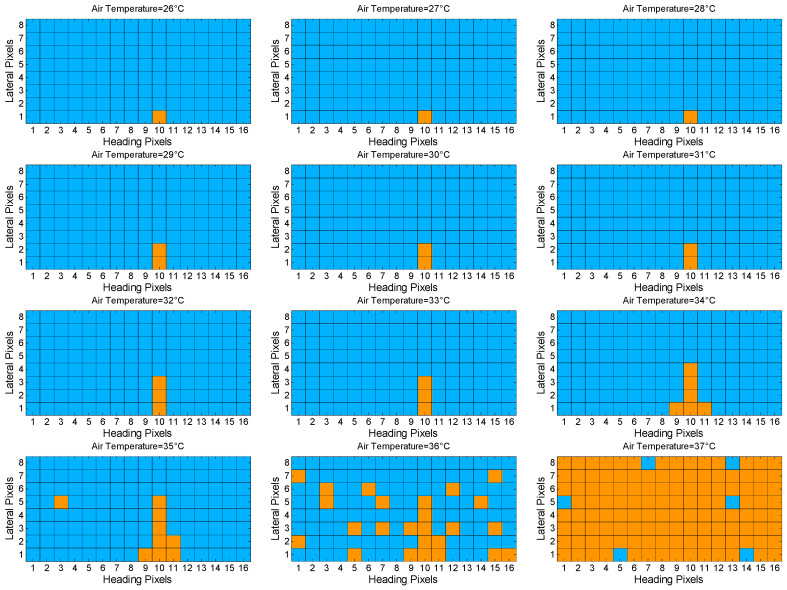
CPD results for a child left in school bus (lying posture, ε = 2).

**Figure 18:**
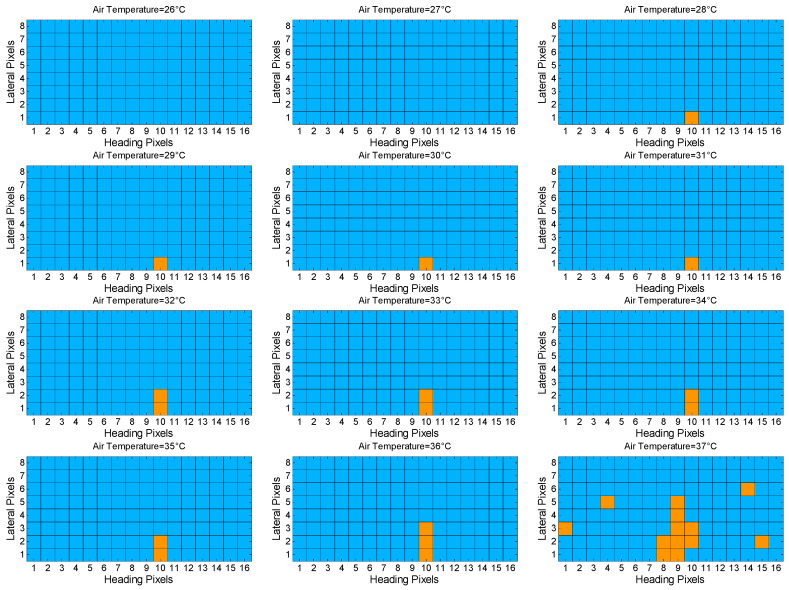
CPD results for a child left in school bus (lying posture, ε = 1).

**Figure 19:**
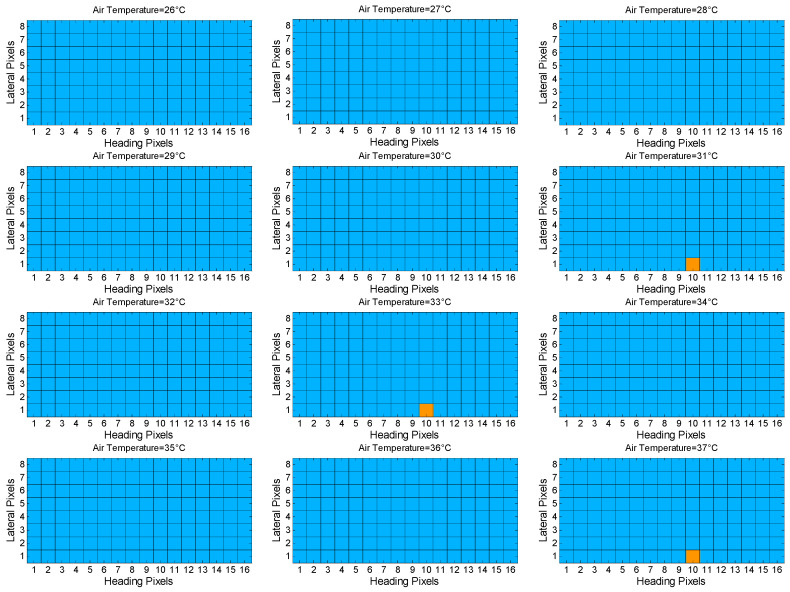
CPD results for a child left in school bus (lying posture, ε = 0.5).

The child presence detection results under different temperature and difference thresholds *ε* for a child remaining in the school bus in a sitting posture are shown in Figure 12, Figure 13, Figure 14, Figure 15 and Figure 16, respectively. The corresponding results for a child in a lying posture are shown in Figure 17, Figure 18 and Figure 19.

In Figure 12, Figure 13, Figure 14, Figure 15, Figure 16, Figure 17, Figure 18 and Figure 19, yellow pixels indicate the presence of a child left behind at that pixel location, while blue pixels indicate the absence of a child left behind. If multiple adjacent pixels appear yellow, it suggests that the child may be active or lying down. If multiple non-adjacent pixels appear yellow, it indicates that the algorithm has produced false detections (false positives). If no pixels are yellow, it indicates that the algorithm has missed a detection (false negative).

Since it has been observed in the sitting posture that the false detection rate becomes high when ε is large, to reduce the workload, only a few smaller values of *ε* are computed for the lying posture.

### 4.3. Analysis of Child Presence Detection Results

#### 4.3.1. Influence of Cabin Ambient Temperature and ε

As shown in Figure 12, when *ε* = 5 and the cabin ambient temperature reaches 29 °C, the algorithm begins to produce false positives. Similarly, from Figure 13, when *ε* = 3 and the cabin ambient temperature reaches 33 °C, false positives start to occur; from Figure 14, when *ε* = 2 and the temperature reaches 35 °C, false positives appear; and from Figure 15, when *ε* = 1 and the temperature reaches 36 °C, false positives begin. Thus, the false-positive rate increases with rising cabin ambient temperature and decreases with decreasing *ε*.

At lower in-cabin ambient temperatures, the human body temperature is significantly higher than the ambient temperature. Consequently, the measured human thermal signal is less influenced by the environment, enabling the algorithm to generate relatively stable and reliable judgments. As the ambient temperature increases, the temperature difference between the human body and the environment gradually decreases. This reduction leads to an increasing number of erroneous judgments, including false positives at pixels where no child is present. Therefore, it is essential to establish a reasonable ambient temperature range within which the algorithm’s decisions are considered valid; results outside this range are deemed invalid.

Furthermore, it can also be observed from Figure 12, Figure 13, Figure 14, Figure 15 and Figure 16 that as ε decreases, the false-positive rate declines, but the false-negative rate increases. The reason is that when ε is too large, the algorithm still judges a child as present even when the deviation between the real-time temperature difference and the presence temperature difference threshold is large, which is clearly unreasonable. Conversely, when *ε* is too small, the algorithm requires the real-time temperature difference to be very close to the presence temperature difference threshold to judge a child as present. However, the infrared array sensors itself has measurement errors, and factors such as air cleanliness and seat material also introduce temperature deviations. When these errors accumulate, even if a child is present, the actual deviation between the real-time temperature difference and presence temperature difference threshold may be large, causing the algorithm to judge no child present due to the overly small *ε*, thereby resulting in false negatives. Consequently, selecting an appropriate *ε* is crucial for the accuracy of the algorithm.

#### 4.3.2. Influence of *P*

Figure 12, Figure 13, Figure 14, Figure 15 and Figure 16 show the experimental results for a child sitting normally in a seat. The results indicate that although false positives occur, the false-negative rate is very low (only when ε = 0.5 do false negatives appear), meaning that child presence can be reliably detected under each ambient temperature. However, in actual school bus scenarios, a child may lie on a seat or lie/squat behind the seat back (as shown in Figure 9). Such postures can reduce the temperature detected by the infrared array sensors or even render the child’s body heat undetectable. If judgments rely solely on the difference between the real-time temperature difference and the presence temperature difference threshold, the results may be biased.

To address this, the proposed algorithm, on the basis of the difference between the real-time temperature difference and the presence temperature difference threshold, further determines whether a child is present by checking whether the number of positive detections at specified cabin ambient temperature points (integer temperature values) exceeds a given presence count threshold *P*. If the number exceeds *P*, a child is considered present; otherwise, the result is attributed to random error. This algorithm design is based on the assumption that children are generally active and do not remain hidden behind seat backs all the time; occasional hiding does not affect the overall judgment. For example, set *P* = 3 and specify the cabin ambient temperatures as 26, 27, …, 33 °C. If the number of positive detections across all these temperature points is 5, the final determination is that a child is present; if the number is 2, the final determination is that no child is present.

Figure 17, Figure 18 and Figure 19 demonstrate that the presence temperature difference threshold, derived solely from the temperature difference between a normally seated child and the cabin ambient temperature, does not account for varying child postures inside the vehicle. When a child lies still, the head temperature is higher than that of areas covered by clothing, and the lying posture reduces the distance from the human body to the sensor and increases the number of pixels occupied. However, the temperature at the pixels occupied by the child is lower than that in the sitting posture. This leads to an increase in the deviation between the real-time temperature difference and the presence temperature difference threshold. If *ε* is set too small, a child in a lying posture may not be detected.

#### 4.3.3. Comparison and Determination of Parameters

The child presence detection error rates under different cabin ambient temperatures, different *ε* and different child postures are shown in Table 1.

The child presence detection accuracy under different ambient temperature ranges, different *ε* and different child postures is shown in Table 2. In Table 2, for the lying posture, if there are two adjacent yellow pixels forming a single connected component, and this component coincides with the experimental position, then the CPD is also considered successful.

According to the results in Table 1 and Table 2, as *ε* decreases, the child presence detection accuracy first increases and then decreases. When *ε* = 1, the detection accuracy is relatively high, suggesting that *ε* = 1 is an appropriate choice. Furthermore, Table 2 shows that when the ambient temperature range (signed as *T*_R_) is 26–33 °C, the child presence detection accuracy is higher than that for the range of 26–37 °C. Therefore, it is recommended to set the temperature detection range to 26–33 °C.

To account for the possibility that a child may not remain in a sitting posture after being left behind (e.g., may lie on a seat), a detection is performed for every 1 °C rise in cabin ambient temperature within the range of 26–33 °C. In this case, requiring all eight detection results to be positive for a final determination of child presence is inappropriate, because the child may change position (e.g., hide behind a seat back) at a specific integer temperature point, resulting in fewer than eight positive detections. Likewise, a single positive detection should not be considered sufficient to confirm child presence, as it may arise from error or interference. Thus, it is necessary to determine a reasonable count threshold *P* for the 8 detection results. Table 3 presents the detection accuracy for different *P*, *ε* and child postures.

A threshold *P* that is too large reduces the child presence detection accuracy, whereas a value that is too small makes the algorithm susceptible to interference and false positives. As shown in Table 3, as *P* decreases, the child presence detection accuracy gradually increases. When *ε* = 1 and *P* = 4, the child presence detection accuracy reaches 100% for both sitting and lying postures. Therefore, *P* = 4 is selected. Specifically, within the ambient temperature range of 26–33 °C, a child presence detection determination is performed for each 1 °C rise in temperature. If four out of the eight determinations indicate child presence, the final output is that a child is present.

Based on the results in Table 1, Table 2 and Table 3, the optimal parameters of the child presence detection algorithm are: *T*_R_ = [26, 33] °C, *ε* = 1 and *P* = 4.

### 4.4. Random Experiments

**Figure 20:**
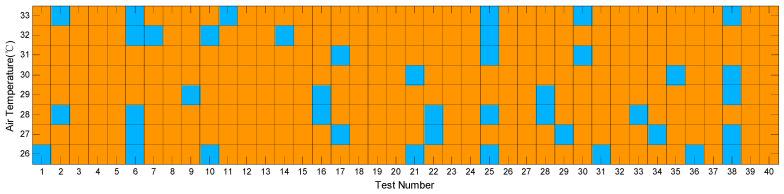
Child presence detection results during random experiments.

**Figure 21:**
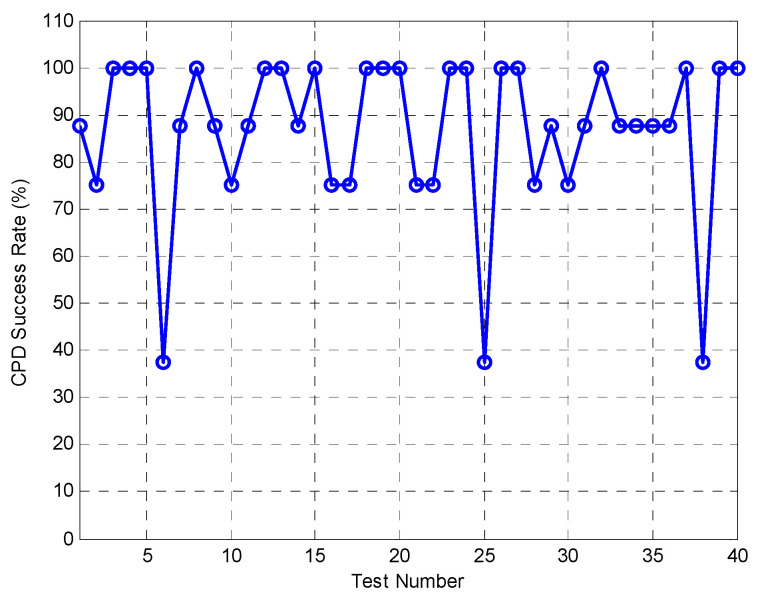
Success rate per random experiment.

**Figure 22:**
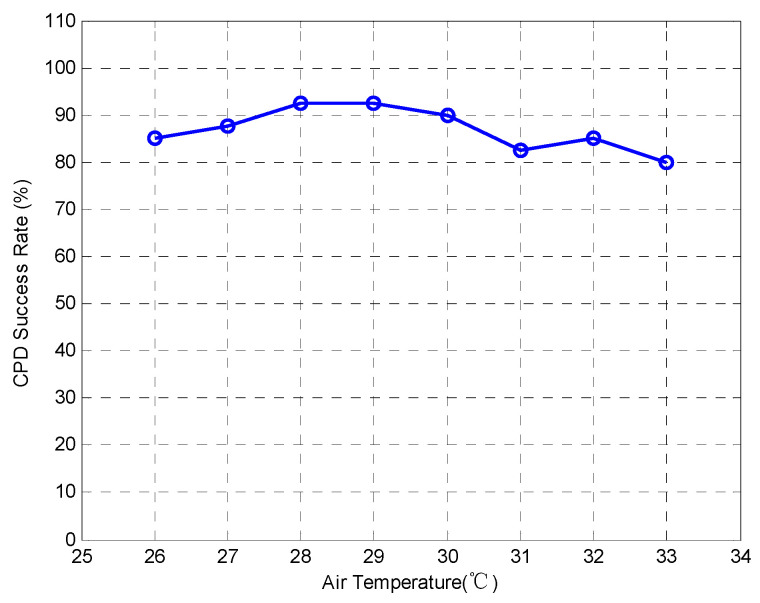
Success rate under different ambient temperatures in random experiments.

The parameter determination in the preceding experiments was performed using fixed seating positions. To further verify the reliability and accuracy of the algorithm, 40 random experiments were conducted, and the results are shown in Figure 20. In these experiments, the upper limit of *T*^(k)^ in Figure 8 of the algorithm flowchart was adjusted to 33 °C. In Figure 20, yellow blocks indicate a detection result of child presence, while blue blocks indicate no child presence. Figure 21 and Figure 22 show the detection success rates under different numbers of detections and different ambient temperatures, respectively.

As shown in Figure 20, Figure 21 and Figure 22, the experimental results are relatively stable at temperatures of 28, 29, and 30 °C, yielding detection success rates of 92.5%, 92.5%, and 90.0%, respectively. At 26 °C and 27 °C, the success rates are 85.0% and 87.5%, which are lower than those observed in the 28–30 °C range. This reduction occurred because the vehicle air conditioner was initially set to 26 °C, and children disembarked in a concentrated manner at the start of the experiments, causing instability in the in-vehicle temperature field and introducing measurement errors. When the ambient temperature exceeded 30 °C, the temperature difference between the human body and the cabin ambient temperature gradually decreased, resulting in reduced algorithm reliability.

In the random experiments, both the positions and postures of the children inside the school bus were randomly determined. A total of 40 experiments were conducted. With *ε* = 1 and *P* = 4, the actual count was less than 4 in 3 experiments, while it was ≥4 in the remaining 37 experiments, yielding an overall detection success rate of 92.5%. This result shows a certain discrepancy compared to the previous fixed-position result. The main reason is that the child was occluded by the seat back, preventing the infrared thermal array sensor from effectively receiving the child’s thermal radiation. Therefore, the proposed technical solution and algorithm for child presence detection still have certain limitations: when a child is in a position or posture that cannot be detected by the sensors (e.g., consistently hiding behind a seat back or lying under a seat), the left-behind child may not be identified. Future research could combine other methods to further improve detection accuracy, such as adding pressure-sensing mats under the seats.

It can also be seen from Figure 20 that although the detection success rate under each individual ambient temperature reached a maximum of 92.5%, the overall success rate is also 92.5%. This outcome arises from the evaluation criterion adopted: within each child presence detection process, a child is considered present if the number of positive detections (i.e., “child present” outcomes) exceeds the preset presence count threshold *P*. This multi-frame confirmation strategy effectively improves the overall detection success rate.

## 5. Discussions

This paper focuses on the method and algorithm for child presence detection in a school bus. The proposed approach adopts two infrared thermal array sensors to capture in-vehicle thermal imaging pixel data and determine the presence of children, achieving satisfactory detection accuracy. Nevertheless, this study still has the following limitations.

(1) Restricted experimental scale. To validate the feasibility and accuracy of the proposed algorithm, only 40 random experiments were conducted in this study, in which a single child was deliberately left alone in the school bus for each trial. For a system that involves children’s life safety, the sample size of 40 random experiments is relatively insufficient. The influence of non-human heat sources on the algorithm’s accuracy also requires further investigation, for example, the presence of hot water bottles, heat-generating electronic devices, seats exposed to prolonged direct sunlight, pets in the vehicle, and so on.
(2) Constrained operating conditions. All experiments in this paper were conducted on a 19-seat children’s school bus, while various types of school buses with different sizes are practically applied in real scenarios. Theoretically, for shorter buses, partial pixel data can be discarded during computation; for longer buses, additional infrared array sensors could be installed, or a rotating mechanism could be added to the existing sensors so that they can scan and fully cover all seating areas. Nevertheless, algorithms tailored to different bus lengths require further investigation and should be verified by additional experiments.
(3) Possible missed detections and false alarms. Although the randomized experiments yielded an overall detection accuracy of 92.5%, this accuracy rate still has scope for enhancement. Missed detections may occur when children hide behind seat backrests or under seats, as their thermal radiation toward the sensors is blocked, thus causing a missed detection. Similarly, heat sources with temperatures close to the human body inside the vehicle may be misjudged as child targets by the algorithm, thereby resulting in false alarms.
(4) Sensor blind spots. The infrared thermal array sensors employed in this study have a field of view of 120° × 25° and are installed on the ceiling of the vehicle, oriented downward. These sensors are inherently incapable of detecting temperature fields that lie outside their viewing range. For instance, children climbing onto the luggage racks inside the school bus cannot be detected by the proposed system.
(5) Dependence on the vehicle configuration. The child presence detection method and algorithm presented in this paper are applicable to children’s school buses that feature symmetric seating arrangements, homogeneous seat materials, and an unmodified interior. For qualified school buses that meet the above conditions, the background temperature and presence temperature only need to be measured once before system deployment. During formal operation, the system integrates calibrated background temperature, presence temperature and real-time temperature data to output child presence detection results via the proposed algorithm. However, if the school bus undergoes major modifications or suffers from uneven seat layouts and inconsistent seat materials after long-term service, the algorithm requires recalibration to ensure detection performance.

In addition to addressing the above limitations, future research will focus on the following areas: (1) establishing a unified experimental platform to enable direct and fair comparison of detection accuracy across different CPD methods under identical test conditions; and (2) implementing multi-sensor data fusion to improve the success rate of detection-for instance, by integrating WiFi-based detection for occluded children, and combining camera-based methods to distinguish non-human heat sources.

## 6. Conclusions

Leaving a child unattended in a school bus can lead to severe consequences, and such incidents occur frequently. To address this issue, this paper investigates detection methods for children left behind in school buses, and draws the following conclusions:

1. A child presence detection (CPD) scheme based on the infrared array sensors is proposed. Two 16 × 4 pixel infrared thermopile array sensors are installed on the bus ceiling, and oriented toward the seating areas. A microcontroller unit (MCU) acquires temperature data in real time, and the corresponding algorithm identifies whether a child has been left in the bus.
2. A child presence detection algorithm is presented. The algorithm requires only a limited number of experiments to obtain the background temperature and presence temperature of the school bus. It computes the real-time temperature difference (between the current temperature and background temperature) and compares it with the presence temperature difference (between the presence temperature and background temperature). If the deviation between these two differences is below a preset threshold, a child is considered present. By performing double differencing, the algorithm effectively mitigates interference arising from variations in material properties at different locations inside the bus, thereby achieving high detection accuracy.
3. The optimal parameters of the algorithm were determined through experiments. Data analysis indicates that when the in-cabin ambient temperature exceeds 33 °C, detection errors increase significantly; thus, the optimal temperature detection range is established as 26–33 °C. A double-difference threshold of *ε* = 1 yields low false-positive and false-negative rates. A presence count threshold of *P* = 4 balances detection accuracy with robustness against transient interference.
4. The feasibility of the algorithm and the rationality of the chosen parameters were verified through experiments. Results from random testing show that, with the proposed algorithm and parameter settings and absence of non-human heat sources, the overall detection success rate reaches 92.5%, representing satisfactory performance. Further improvements in success rate can be achieved by combining this method with other detection technologies.

## Figures and Tables

**Table 1:** CPD error rate (%) under different temperatures *ε* and child postures.

Temperature (°C)	Sitting					Lying		
	*ε* = 5	*ε* = 3	*ε* = 2	*ε* = 1	*ε* = 0.5	*ε* = 2	*ε* = 1	*ε* = 0.5
26	0.00	0.00	0.00	0.00	0.00	0.00	−0.78	−0.78
27	0.00	0.00	0.00	0.00	0.00	0.00	−0.78	−0.78
28	0.00	0.00	0.00	0.00	0.00	0.00	0.00	−0.78
29	+0.78	0.00	0.00	0.00	−0.78	+0.78	0.00	−0.78
30	+2.34	0.00	0.00	0.00	−0.78	+0.78	0.00	−0.78
31	+2.34	0.00	0.00	0.00	0.00	+0.78	0.00	0.00
32	+4.69	0.00	0.00	0.00	−0.78	+1.56	+0.78	−0.78
33	+13.28	+0.78	0.00	0.00	−0.78	+1.56	+0.78	0.00
34	+99.22	+4.69	0.00	0.00	−0.78	+3.91	+0.78	−0.78
35	+99.22	+7.81	+1.56	0.00	0.00	+6.25	+0.78	−0.78
36	+99.22	+96.88	+25.00	+0.78	−0.78	+18.75	+1.56	−0.78
37	+99.22	+99.22	+92.97	+10.94	0.78	+94.53	+9.38	0.00

Note: “+” indicates a false positive, and “−” indicates a false negative.

**Table 2:** CPD accuracy (%) under different temperature ranges, *ε* and child postures.

Temperature Ranges (°C)	Sitting					Lying		
	*ε* = 5	*ε* = 3	*ε* = 2	*ε* = 1	*ε* = 0.5	*ε* = 2	*ε* = 1	*ε* = 0.5
[26, 33]	37.5	87.5	100.0	100.0	50.0	75.0	75.0	25.0
[26, 37]	25.0	58.3	75.0	83.3	41.7	50.0	66.7	25.0

**Table 3:** CPD accuracy (%) for different *P*, *ε* and child postures.

*P*	Sitting					Lying		
	*ε* = 5	*ε* = 3	*ε* = 2	*ε* = 1	*ε* = 0.5	*ε* = 2	*ε* = 1	*ε* = 0.5
8	37.50	87.50	100.00	100.00	50.00	75.00	75.00	25.00
7	42.86	100.00	100.00	100.00	57.14	85.71	85.71	28.57
6	50.00	100.00	100.00	100.00	66.67	100.00	100.00	33.33
5	60.00	100.00	100.00	100.00	80.00	100.00	100.00	40.00
4	75.00	100.00	100.00	100.00	100.00	100.00	100.00	50.00
3	100.00	100.00	100.00	100.00	100.00	100.00	100.00	66.67
2	100.00	100.00	100.00	100.00	100.00	100.00	100.00	100.00
1	100.00	100.00	100.00	100.00	100.00	100.00	100.00	100.00

## Data Availability

The original contributions presented in this study are included in the article. Further inquiries can be directed to the corresponding author.
